# Autonomy and Submissiveness as Cognitive and Cultural Factors Influencing Eating Disorders in Italy and Sweden: An Exploratory Study

**DOI:** 10.5964/ejop.v11i2.902

**Published:** 2015-05-29

**Authors:** Sandra Sassaroli, Guido Veronese, Lauri Nevonen, Francesca Fiore, Franceso Centorame, Ettore Favaretto, Giovanni Maria Ruggiero

**Affiliations:** a Studi Cognitivi, Post-graduate Psychotherapy School, Milan, Italy; b Department of Human Sciences, University of Milano-Bicocca, Milan, Italy; c School of Health and Medical Sciences, Örebro University, Örebro, Sweden; d Psicoterapia Cognitiva e Ricerca, Post-graduate Psychotherapy School, Milan, Italy; e Azienda Sanitaria Bolzano, Bolzano, Italy; University of South Wales, Newport, United Kingdom

**Keywords:** eating disorders, culture, collectivism, individualism

## Abstract

The aim of this exploratory study was to investigate the correlation between cultural and psychological factors in relation to predicting eating disorders in two different non-clinical Italian (n = 61) and Swedish (n = 31) female populations, thought to have different cultures and lifestyles. The Swedish sample would reflect an emancipated model of women pursuing autonomy and freedom but also an ideal of thinness, while the Italian sample would reflect a difficult transition from traditional submissiveness to modern autonomy. Both groups completed self-report instruments assessing cultural values (e.g., collectivism and individualism) and features of eating disorders (e.g., drive for thinness, bulimia, body dissatisfaction, self-esteem, parental criticism and perfectionism). Swedish women were found to display higher levels of bulimia, perfectionism, and individualism than Italian women, while regression analysis showed that in the Italian sample high levels of collectivism were correlated with measures of EDs. The results support the hypothesis that EDs are linked with both modern values of autonomy, independence and emancipation, and situations of cultural transition in which women are simultaneously exposed to traditional models of submission and opportunities for emancipation and autonomy.

## Introduction

Cultural attitudes and beliefs have been shown to be significant contributing factors in the development of eating disorders (EDs). Many researchers and clinicians consider the ideal of slim female beauty promoted by the media and pursued in North American and European countries since the 1960s to be a cultural determinant of EDs (Garner, Garfinkel, Schwartz, & Thompson, 1980; Hesse-Biber & Carter, 2005; Hesse-Biber, Leavy, Quinn, & Zoino, 2006; Silverstein, Perdue, Peterson, & Kelly, 1986; Wiseman, Gray, Mosimann, & Ahrens, 1992). This ideal of thin female beauty is presumably linked to increasing levels of urbanization and industrialization (Gordon, 1988). Hence, the rise in EDs in Western countries may be attributed at least in part to the modern cultural shift towards individual autonomy and freedom and the rejection of a traditional lifestyle based on patriarchal and familial values (Kashubeck-West & Tagger, 2012; Katzman & Lee, 1997; Nasser, 1988).

Nevertheless, this notion of modernization as a risk factor for EDs may be insufficient. A second line of research has explored the alternative hypothesis that ED may not only depend on the modern ideal of thinness but also on emotional suffering linked to the cultural clash between increased levels of urbanization and modernization and the persistence of traditional patriarchal and familial values (Nasser, 1988). Traditional society would be characterized by submission, adherence to communitarian goals, religiously-motivated respect for stable social and sexual roles, confidence in higher non-personal “spiritual” meanings, and dysfunctional acceptance of hierarchical organizations. In contrast, a modern individualistic mentality would lead to the pursuit of personal autonomy and self-confidence, the setting of personal goals, and the building of functional relationships among peers to achieve concrete aims (Nasser, 1988; Ruggiero, 2003).

According to Katzman and Lee (1997), it is plausible to suggest that in a changing cultural environment women may develop an ED not only as a result of dieting, and weight and fat phobia, but also as a reaction to sociocultural disconnection and transition. The question then becomes, do women affected by EDs fear modern autonomy and pursue asceticism, rejecting the ideal of the body, the material world and the personal self? Alternatively, are women with EDs pursuing perfectionism, autonomy, self-control, a sense of triumph over the limitations of the body, and social assertiveness?

An environment in which the above-mentioned condition of cultural transition can be found is Southern Italy. Sociological studies have shown that the social status of women in Southern Italy is undergoing a transition (Leccardi, 1995). In particular, Southern Italian adolescents live in a society in which the era of the large, patriarchal family, made up of several siblings and other kin of varying degrees, is coming to an end. The modern tendency is for nuclear families with two or three children, yet there is a conspicuous lack of a social group for teenagers that would allow them to develop an independent personality outside the family. The girls in this society are trapped in the "small" nuclear family (Leccardi, 1995). Up to 1998 Italy had a weak tradition of implementing gender equality policies (Rubery, 2002; Villa, 2001) and, despite marked progress in gender mainstreaming since 1999, in 2013 the Italian female employment rate was still significantly lower than the average European and Swedish rates: 47.8% (Italy), 58.6% (Europe) and 71.8% (Sweden) (Organization for Economic Cooperation and Development [OECD], 2013). What is more, if we consider Southern Italian women only, the employment rate is even lower: 20.7% (Istituto Nazionale di Statistica [ISTAT], 2013). Consequently Southern Italian women develop a lower drive for autonomy and independence that could be hypothetically linked with lower sensitivity to models of female beauty (Ruggiero, 2003). However, Southern Italian women also suffer because of internal familial conflict between exposure to the modern models of consumerism and gender equality as represented by the mass media (Ruggiero, 2003) and a daily reality of submission and difficult access to employment. Such conflicts may act as a cultural pathogenic factor in EDs that may be just as powerful as exposure to modern values of thin female beauty (Katzman & Lee, 1997).

Swedish women on the other hand live in a society with a high level and sense of autonomy. Sweden has one of the highest levels of gender equality in the world (OECD, 2013). This is based on the belief that when women and men share power and influence equally it leads to a more just and democratic society (Borchorst & Siim, 2008; Hernes, 1987). An advanced welfare system makes it easier for both sexes to balance their work and family life (Hofstede, Hofstede, & Minkov, 2010). Thanks also to a well-developed ‘state feminism’ that results in a particularly woman-friendly welfare state (Hernes, 1987), Swedish women face fewer obstacles in developing their personal autonomy and making independent choices in relation to their personal, social and affective lives or career ambitions (Borchorst & Siim, 2008).

### Aim of the Study and Hypotheses Tested

In sum, the scientific literature suggests that EDs are underpinned by both sociocultural and psychological factors. Within the sociocultural set, a first stream of studies exclusively related EDs to a cultural ideal of thinness and modern values of autonomy, individualism and independence; a second wave of studies added another set of cultural variables related to family conflicts fuelled by cultural contrasts between traditional values of submissive adherence to hierarchy and authority and modern values of autonomy and independence.

The current research aimed to explore the effects of the two above-mentioned sets of cultural factors associated with EDs, by testing both the model in which the first set of variables is sufficient to create a predisposition for EDs (Hypothesis 1) and the model proposing that both the sets of cultural factors contribute to the psychopathological mechanism, in other words that thinness may be pursued not only as a sign of autonomy and perfection but also as a protest against persistent submission and perceived control during cultural transition (Hypothesis 2).

## Method

### Participants

Ninety-six women whom screening had shown not to be affected by EDs (see procedures) participated in the study. Sixty-one were from Italy (mean age 21.72 ± 1.99 years) and 35 from Sweden (mean age 21.51 ± 2.08 years). All were high school graduates without a university degree. Three Italian participants had been unemployed in the six months prior to the study. Fifty-one of the Italian women defined themselves as “Catholic”; 14 Swedish individuals defined themselves as “Protestant” and two as “Catholic”; while all other participants declared no religious affiliation.

### Instruments

In order to measure cultural factors we used the Individualism–Collectivism Scale (ICS). The ICS was developed by Triandis (2001) with the aim of assessing the construct of individualism–collectivism at the individual level. Participants rate how strongly they agree or disagree with each statement on a 5-point scale, ranging from 1 (strongly disagree) to 5 (strongly agree). The ICS is composed of 16 items assessing individualism and 16 items assessing collectivism. These items are subdivided into four categories: horizontal individualism (HI), vertical individualism (VI), horizontal collectivism (HC), and vertical collectivism (VC). VI is deﬁned as self-interest and competition, HI by independence and autonomy, VC is deﬁned as giving priority to group goals over individual goals and having respect for elders and persons in authority, and HC is characterized by relationship-orientation and harmony. The first author translated the ICS into Italian. The second author translated the questionnaire into Swedish. Both versions were then back-translated into English by native English speakers from the US who were unfamiliar with the tool. An English language teacher from the US then compared the back-translations from the Italian and Swedish and found no meaningful discrepancies between them (Romano Denaro, June 23, 2004, personal communication).

As measures of EDs, we chose the drive for thinness (DT-EDI), bulimia (B-EDI), and body dissatisfaction (BD-EDI) subscales of the Eating Disorders Inventory–Version 3 (EDI-3, Garner, 2004). The DT-EDI, in particular, assesses a key component of EDs, and is useful in screening for them (Abood & Black, 2000; Engström et al., 1999). According to Garner (1991, 2004), the DT-EDI subscale assesses excessive concern with dieting, preoccupation with weight, and fear of weight gain. Polivy and Herman (1987) found that high DT-EDI scores for college women reflected a preoccupation with weight that was as severe as that of individuals with an ED. The drive for thinness construct is based on the clinical theorizing of Hilde Bruch (1973, 1982) and Gerald Russell (1970). For the purposes of the current study, the first author translated the questionnaire into Italian. The Italian version of the questionnaire was then back-translated into English by a native English speaker from the US who was unfamiliar with the tool. The author of the EDI compared the original version with the back-translated version of the instrument and did not find any significant differences (D.M. Garner, e-mail communication to Brenda VanAntwerp, January 15, 1997). The second author translated the questionnaire into Swedish. This version was then back-translated into English by a native of the US who was unfamiliar with the tool. An English language teacher from the US compared the back-translations from Italian and Swedish of the DT-EDI, B-EDI, and BD-EDI subscales and found no meaningful discrepancies between them (Romano Denaro, June 23, 2004, personal communication).

The Rosenberg Self-Esteem Scale (RSES; Rosenberg, 1965) assesses global self-esteem and sense of self-worth. It is a 10-item instrument. Items are rated on a four-point Likert scale, ranging from 3 (strongly agree) to 0 (strongly disagree). A significant body of research has shown the RSES to have satisfactory reliability and validity when used with adults (Corcoran & Fisher, 2000, p. 610). The first author translated the RSES into Italian. The second author translated the questionnaire into Swedish. These versions were then back-translated into English by a native of the US who was unfamiliar with the tool. An English language teacher from the US compared the back-translations of the RSES from Italian and Swedish and found no meaningful discrepancies (Romano Denaro, June 23, 2004, personal communication).

Concern over Mistakes (CM-FMPS) is a 9-item subscale of the Frost Multidimensional Perfectionism Scale (FMPS; Frost, Marten, Lahart, & Rosenblate, 1990). The FMPS is a 35-item self-report questionnaire comprising six subscales: Concern over Mistakes, Doubts about Actions, Personal Standards, Parental Expectations, Parental Criticism, and Organization. The CM-FMPS assesses the aspect of perfectionism that is most closely associated with anxious and obsessive psychopathology (DiBartolo, Li, & Frost, 2008). Specifically, the Concern over Mistakes subscale assesses negative reactions to mistakes and perceptions of even minor mistakes as failure. This measure has proven internal consistency, high test-retest reliability, and a strong capacity to discriminate individuals affected by obsessive compulsive disorder, panic disorder and social anxiety from non-clinical subjects (Frost et al., 1990).

The first author translated the FMPS into Italian. This version was then back-translated into English by a native of the US who was unfamiliar with the tool. One of the authors of the MPS compared the original version and the back-translated version and did not find any meaningful differences (Randy Frost, December 29, 2004, e-mail communication). The second author translated the questionnaire into Swedish. This version was then back-translated into English by a native of the US who was unfamiliar with the tool. An English language teacher from the US compared the back-translations from Italian and Swedish and found no meaningful discrepancies (Romano Denaro, June 23, 2004, personal communication).

### Procedures

We recruited purposive convenience samples in different workplaces in Italy and Sweden. Italian and Swedish psychologists assessed the demographic data and details of past or current psychological and/or psychopharmacological treatments supplied by the participants. The psychologists first administered the Structured Clinical Interview for DSM-IV Axis 1 Disorders (SCID-I; First, Spitzer, Gibbon, & Williams, 1997) as a screening measure for eating, anxiety or mood disorders. No participant met the screening measure criteria for diagnosis with an ED and, consequently, all the participants were included in the database. Further inclusion criteria included a minimum age of 18 years and the ability to adequately comprehend the written national language. All participants signed an informed consent form, were allowed to withdraw from the study whenever they wanted, and were not remunerated for their participation.

This research followed the American Psychological Association’s (2010) Ethical Principles of Psychologists and Code of Conduct and was approved by the Ethical Board of the Milano-Bicocca University and of the Studi Cognitivi Post-graduate Psychotherapy School.

### Controlled Psychological Variables

In order to test the influence of cultural factors on EDs, it was important to control for the effects of the well-known psychological correlates of EDs. Perfectionism and low self-esteem are the most powerful psychological correlates of EDs (Stice, 2002). Individuals with an ED are frequently oppressed by persistent and vague feelings of not being sufficiently qualified, competent, or suited to the demands of life; spend a lot of time worrying about these negative self-evaluations (Fairburn & Harrison, 2003; Sassaroli et al., 2005; Vitousek & Hollon, 1990); and strive for perfection either in terms of pursuing ideals of thinness and bodily appearance or in other aspects of life (Bardone-Cone, 2007; Boone, Soenens, Vansteenkiste, & Braet, 2012; Castiglioni, Faccio, Veronese, & Bell, 2013; Castiglioni, Pepe, Gandino, & Veronese, 2013; Sassaroli et al., 2011; Sassaroli et al., 2005).

### Data Analysis

We conducted an analysis of variance (ANOVA) to test for significant differences between the Italian and Swedish samples (Hypothesis 1). Linear regressions were used to test the relationships among ED measures, psychological measures and cultural measures (Hypothesis 2).

## Results

### Preliminary Analyses

Regarding the internal consistency of the instruments, Cronbach’s alpha values for the ICS subscales were: 0.75 among Southern Italian women and 0.77 among Swedish women for the VI subscale; 0.71 among Southern Italian women and 0.78 among Swedish women for the HI subscale; 0.76 among Southern Italian women and 0.81 among Swedish women for the VC subscale; 0.68 among Southern Italian women and 0.78 among Swedish women for the HC subscale. Cronbach’s alpha were 0.071 among Southern Italian women and 0.73 among Swedish women for the CM-FMPS; 0.66 among Southern Italian women and 0.79 among Swedish women for the DT-EDI; 0.072 among Southern Italian women and 0.73 among Swedish women for the B-EDI; 0.81 among Southern Italian women and 0.77 among Swedish women for the BD-EDI; and 0.69 among Southern Italian women and 0.78 among Swedish women for the RSES.

### Italy-Sweden Comparison

The Swedish sample obtained significantly higher mean scores than the Italian sample on one of the measures of ED (B-EDI), two psychological scales (CM-FMPS and PC-FMPS), and one cultural scale (HI). The Italian sample obtained higher mean scores on two cultural scales (HC and VC). No significant differences were found in respect of DT-EDI and BD-EDI, RSES, or VI. Table 1 reports these results in detail.

**Table 1:** Comparison of Mean Scores of Italian and Swedish Samples

Variable	Italy (*n* = 61)	Sweden (*n* = 35)	ANOVA	
	*M* (*SD*)	*M* (*SD*)	*F (df =181)*	*Effect Size*
Drive for Thinness	3.33 (4.80)	4.37 (2.56)	0.655	.345
Bulimia	.93 (1.41)	7.34 (3.41)	9.802***	.234
Body Dissatisfaction	9.21 (5.12)	10.20 (3.62)	2.134	.463
Self-esteem	30.52 (4.72)	28.51 (5.49)	0.858	.278
Concern over Mistakes	18.84 (5.95)	22.69 (8.53)	1.955*	.367
Parental Criticism	8.08 (3.06)	14.66 (4.04)	3.103***	.456
Horizontal Collectivism	94.91 (9.51)	82.91 (12.90)	3.312***	.567
Vertical Collectivism	.82 (16.26)	-9.91 (17.31)	4.392**	.135
Horizontal Individualism	7.44 (17.00)	24.40 (10.30)	6.107***	.589
Vertical Individualism	70.07 (10.16)	70.40 (11.02)	8.502	.456

*Note.* *p < .05. **p < .01. ***p < .001.

In sum, comparison of the two samples revealed lower levels of collectivism and higher levels of individualism, perfectionism, and bulimia in the Swedish sample with respect to the Italian sample.

### Relationship Between Cultural Variables and Measures of Eating Disorders

Based on these results we wished to test whether there was a relationship among the variables. We therefore conducted a series of linear regressions on the Italian and Swedish groups separately.

The results indicated that in the Italian sample VC (independent variable) predicted B (dependent variable) (*R^2^* = .52; β = 0.217; *t* = 6.614; *p* < .05); while in the Swedish sample VC (independent variable) predicted DT (dependent variable) (*R^2^* = .63; β = 0.70; *t* = 2.62; *p* < .05), and HC (independent variable) predicted BD (dependent variable) (*R^2^* = .67; β = 0.052; *t* = 2.68; *p* < .05).

## Discussion

The fact that Swedish women tended to display higher levels of bulimia, perfectionism and individualism than Italian women supports the argument (Hypothesis 1) linking EDs to the cultural values of autonomy, independence, and emancipation that are typical of modern individualistic and affluent societies (Kashubeck-West & Tagger, 2012; Katzman & Lee, 1997; Nasser, 1988; Ruggiero, 2003).

On the other hand, the regression analysis showed that in the Italian sample high levels of collectivism were correlated with measures of EDs. This result supports the hypothesis linking EDs with cultural transitions during which women are exposed to both traditional models of submission and opportunities for emancipation and autonomy (Hypothesis 2). This is in line with the cultural model proposed by Katzman and Lee (1997), which states that modern women’s high level of cultural autonomy of may be related to a strong need for self-assertiveness, and, in people who are lacking in self-confidence and unable to meet these high standards, this may lead to high levels of anxiety, emotional confusion, social alienation, and cultural disconnection. The challenge posed by the increased sense of autonomy, self-confidence and the pursuit of personal goals may generate insecurity and the need for vertical values of submission and dependence. In turn, women may express this emotional suffering by adopting maladaptive eating habits, such as restricted eating and bulimic behaviours (Katzman & Lee, 1997).

It is likely not by chance that this line of research has mainly investigated cultures outside of Northern Europe and Northern America. For example, it seems that women in Asia may practice self-starvation in order to achieve self-determination when confronted with ambivalent cultural demands, with minimal importance attributable to weight and shape concerns (Lee, 2001). Anorexic and bulimic Japanese women report significantly lower rates of drive for thinness than their respective North American counterparts (Pike & Mizushima, 2005). Whereas in Western culture thinness is often associated with having power and control, which in turn are associated with happiness, the Japanese pursuit of thinness seems to serve as a strategy for postponing growing up and taking on the related responsibilities. There is evidence to suggest that body dissatisfaction plays a more significant part among American women than among Japanese women in the development of eating disorders (Mukai, Kambara, & Sasaki, 1998). These findings imply that it is mistaken to universally equate body image disturbance with weight and shape concerns. It appears that other dimensions need to be explored in order to construct a more culturally sensitive understanding of body dissatisfaction (Pike & Borovoy, 2004).

Furthermore, Katsounari and Zeeni (2012) compared Lebanese and Cypriot female students, finding that, while Cypriot students were more preoccupied with their weight, Lebanese students received higher emotional and external eating scores. These findings suggest that in Lebanese culture, eating dysfunction among women may be due to responsiveness to external and emotional cues, while in Cypriot culture it may be due to an excessive preoccupation with weight fuelled by sociocultural agents. In conclusion, this study seems to support the hypothesis that in non-Western cultures EDs are less directly linked to the pursuit of thinness, and more directly related to emotional suffering.

Katzman and Lee’s model seems to be applicable to the Italian sample. On the other hand, Swedish women seem to show that higher values of ED measures may also be related to individualism. These results suggest a connection between the need for increased personal autonomy and a personal ideal of slim female beauty that has been promoted by the media and pursued by women in many North American and European countries since the 1960s (Altabe & Thompson, 1994; Cattarin & Thompson, 1994).

To summarize our findings, therefore, the Italian data provided strong evidence in favour of Hypothesis 2, which was borne out by the results of the regression analyses, while the Swedish data yielded weaker support for Hypothesis 1. In conclusion, the strength of this study is that it provides confirmation for both the models under enquiry, namely the earlier model linking EDs to modern individualism and the later one in which EDs are viewed as influenced by a cultural clash between traditional and modern values. The prevalence of a given model appears to depend on environmental circumstances: in fully individualistic cultural settings such as Sweden, the first model seems to be the dominant one; in cultural contexts in transition such as Southern Italy, we may observe the second model in action. A key clinical implication is that in order to enhance their resilience against EDs, individuals need to fully and functionally integrate modern and traditional values, an integration that can overcome both acritical adherence to modern individualism and destructive culture clashes between modernity and traditionalism (Kashubeck-West & Tagger, 2012; Ruggiero, 2003). Of course, it is necessary for these research outcomes to be replicated by future studies.

The study displays a number of limitations, the most important of which are the non-clinical nature of the sample and the use of self-report instruments. In addition, both the research design and our interpretation of the findings rely on the assumption that there were cultural differences between the Italian and the Swedish samples.
